# c‐MYB‐ and PGC1a‐dependent metabolic switch induced by MYBBP1A loss in renal cancer

**DOI:** 10.1002/1878-0261.12499

**Published:** 2019-06-11

**Authors:** Blanca Felipe‐Abrio, Eva M. Verdugo‐Sivianes, Amancio Carnero

**Affiliations:** ^1^ Instituto de Biomedicina de Sevilla (IBIS), Hospital Universitario Virgen del Rocío (HUVR), Consejo Superior de Investigaciones Científicas Universidad de Sevilla Spain; ^2^ CIBER de Cáncer Instituto de Salud Carlos III Madrid Spain

**Keywords:** c‐MYB, metabolism, MYBBP1A, PGC1α, renal cancer

## Abstract

The tumor microenvironment may alter the original tumorigenic potential of tumor cells. Under harsh environmental conditions, genetic alterations conferring selective advantages may initiate the growth of tumor subclones, providing new opportunities for these tumors to grow. We performed a genetic loss‐of‐function screen to identify genetic alterations able to promote tumor cell growth in the absence of glucose. We identified that downregulation of MYBBP1A increases tumorigenic properties under nonpermissive conditions. MYBBP1A downregulation simultaneously activates PGC1α, directly by alleviating direct repression and indirectly by increasing *PGC1α* mRNA levels through c‐MYB, leading to a metabolic switch from glycolysis to OXPHOS and increased tumorigenesis in low‐glucose microenvironments. We have also identified reduced *MYBBP1A* expression in human renal tumor samples, which show high expression levels of genes involved in oxidative metabolism. In summary, our data support the role of MYBBP1A as a tumor suppressor by regulating c‐MYB and PGC1α. Therefore, loss of MYBBP1A increases adaptability spanning of tumors through metabolic switch.

**Abbreviations**
2DG: 2‐deoxy‐d‐glucose
ccRCC: clear cell renal cell carcinoma
chRCC: chromophobe renal cell carcinoma
GAPDH: glyceraldehyde‐3‐phosphate dehydrogenase
MYB: avian myeloblastosis viral oncogene homolog
MYBBP1A: MYB‐binding protein 1a
NRD: negative regulatory domain
PPARGC1A/PGC1a: peroxisome proliferator‐activated receptor‐gamma coactivator 1/PPAR‐gamma coactivator 1‐alpha
pRCC: papillary renal cell carcinoma
ROS: reactive oxygen species
shRNA: short hairpin ribonucleic acid
TCGA: The Cancer Genome Atlas
VHL: von Hippel–Lindau

## 1 Introduction

The host microenvironment in which tumor cells are located may alter the original tumorigenic potential of these cells. Specific microenvironmental conditions may select for the most robust clone, allowing it to hierarchically evolve, which generates a large tumor mass. Conversely, the same cell located in a nonpermissive environment may not contribute to tumor growth. Impaired vascularization during early and later stages of tumor growth causes an altered microenvironment that lacks oxygen and nutrients, which greatly impairs the development of tumors (Carnero and Lleonart, 2016). In these cases, hypoxia has emerged as an essential factor for tumor physiology by promoting tumor initiation, progression, and resistance to therapy. Hypoxia has been related to changes in cellular metabolism by providing an alternative source of nutrients at a very high cost of glucose consumption (Kroemer and Pouyssegur, 2008). Even in the presence of oxygen, cancer cells switch from generating ATP by the highly energy‐efficient process of oxidative phosphorylation to the much less efficient process of glycolysis. Undoubtedly, we now know that this metabolic switch, called the Warburg effect, is an important feature of cancer cells (Warburg, 1956). Aerobic glycolysis facilitates cellular transformation by producing high levels of glycolytic intermediates that cancer cells need for the biosynthesis of lipids, amino acids, and nucleic acids (Cairns *et al.*, 2011). However, before neovascularization replenishes a tumor with new blood vessels, the tumor is subjected to very low glucose conditions. Under these critical environmental conditions, genetic alterations that provide selective advantages may initiate the growth of tumor subclones, providing new opportunities for these tumors. Heterogenetic tumors made up of different clones are able to adapt to different environmental conditions, providing the basis for therapy resistance.

The peroxisome proliferator‐activated receptor γ coactivator (PGC1α) is a known regulator of mitochondrial oxidative metabolism. PGC1α was first discovered as a coactivator of the adipogenic nuclear receptor PPARγ (Puigserver *et al.*, 1998). By binding to several transcription factors and nuclear receptors, PGC1α induces the activation of genes involved in the tricarboxylic acid cycle (TCA cycle), oxidative phosphorylation, fatty acid beta‐oxidation, mitochondrial biogenesis, and mitochondrial reactive oxygen species (ROS) detoxification (Scarpulla, 2011; Ventura‐Clapier *et al.*, 2008). Furthermore, PGC1α suppresses glycolysis in melanoma tumors (Lim *et al.*, 2014), and it has been proposed as a potential suppressor of glycolysis through NRF‐1 control of von Hippel–Lindau (VHL; Scarpulla, 2011). Therefore, PGC1α is a transcription factor coactivator that influences the majority of cellular metabolic pathways and several crucial aspects of energy metabolism. The expression of *PGC1*α is rapidly induced in situations that demand mitochondrial production of heat or ATP, such as cold exposure, short‐term exercise, and fasting (Kelly and Scarpulla, 2004). Abnormal expression of PGC1α is associated with several chronic diseases, and in recent years, it has been shown to be a critical controller of cancer development. The characteristic traits of PGC1α in maintaining metabolic homeostasis promote cancer cell survival and tumor metastasis in harsh microenvironments (Tan *et al.*, 2016).

The 160‐kDa MYB‐binding protein 1A (MYBBP1A, p160) is a repressor of PGC1α and can be a key regulator of metabolic processes. MYBBP1A interacts with the negative regulatory domain (NRD) of PGC1α and reduces its ability to stimulate mitochondrial respiration and electron transport system‐related gene expression. This negative regulation is removed when PGC1α is phosphorylated by p38 MAPK (Fan *et al.*, 2004). The ubiquitously expressed MYBBP1A was originally found to interact with the c‐MYB oncogene product. MYBBP1A binds to the leucine zipper motif in the NRD of c‐MYB (Tavner *et al.*, 1998). MYBBP1A also binds to several other transcription factors, such as p53, enhancing its acetylation and accumulation (Kumazawa *et al.*, 2015; Ono *et al.*, 2014).

MYBBP1A is located on chromosome 17p13.3, which loses heterozygosity at high frequency (up to 50–80%) in different malignancies (Keough *et al.*, 1999). Besides its role as a nucleolar transcriptional regulator, MYBBP1A is essential in mice prior to blastocyst formation, is involved at premitotic level, and may display tumor suppressor activity (Mori *et al.*, 2012). In addition, MYBBP1A is regulated by the VHL tumor suppressor (Lai *et al.*, 2011), which regulates MYBBP1A degradation in an iron‐ and proteasome‐dependent manner. Therefore, MYBBP1A could also be involved in the metabolic plasticity of cancer cells. However, there is not completely understood the molecular mechanism through which MYBBP1A would regulate the tumor metabolism.

In this work, we performed a genetic loss‐of‐function screen to identify genetic alterations that promote growth in the absence of glucose. We identified that downregulation of MYBBP1A switches glycolytic metabolism to oxidative phosphorylation (OXPHOS) by activating PGC1α directly and indirectly through c‐MYB activation. Interestingly, only renal cancer cell lines that express high levels of c‐MYB and do not express pVHL can take advantage of the cellular metabolism switch and increase tumorigenesis. We also analyzed public transcriptome databases and found that reduced *MYBBP1A* expression is correlated with high expression of genes involved in oxidative metabolism in a percentage of cases. Our data strongly support the role of MYBBP1A as a tumor suppressor via regulation of c‐MYB and PGC1α.

## 2 Materials and methods

### 2.1 Cell culture

All human renal tumor cell lines were obtained from Cell Line Service (CLS) (Eppelheim, Germany), which performed the authentication test. ACHN and A498 were maintained in DMEM (AQmedia; Sigma, St. Louis, MO, USA). 786‐0 and CaKi‐1 were maintained in RPMI 1640 (AQmedia; Sigma). For the surrogated assays, RPMI 1640 without glucose (Gibco, Paisley, UK) and DMEM without glucose (Gibco) were used. For metabolic assays, RPMI (Bio‐West, Nuaillé, France) and DMEM (Corning, Corning, NY, USA) without l‐glutamine, sodium pyruvate, and glucose were used. GlutaMAX (Gibco) or glucose was added depending on the experimental requirements. All media were supplemented with 10% FBS (Gibco), penicillin, streptomycin, and fungizone (Sigma). Normoxia is considered at 21%. Hypoxia is defined usually at 3% oxygen. When 1% or 5% oxygen is used for hypoxia, this is specified in the experiment.

### 2.2 Genetic loss‐of‐function screen

Library generation was performed as described previously (Leal *et al.*, 2008). Transduction of the library and recovery of the proviruses were performed as previously described by Carnero and coworkers (Leal *et al.*, 2008). For selection, NIH3T3 cells were cultured in media without glucose.

### 2.3 RNA array hybridization

Cancer profiling array membranes (BD Biosciences, Franklin Lanes, NJ, USA) were prehybridized with ExpressHyb hybridization solution for 4 h at 65 °C. The appropriate probe was then labeled by PCR with 50 °C of redivue dCTP32 (Amersham, Buckinghamshire, UK). The labeled probe was then purified from free hot nucleotides with a sepharose G‐50 column Nick (Amersham). The purified probe was then denatured for 3 min at 100 °C and added to the hybridization solution. The hybridization was performed overnight at 65 °C. Then, the membrane was washed at 65 °C two times with 2× SSPE, 0.1% SDS; once with 1× SSPE, 0.1% SDS; and once more with 0.1× SSPE, 0.1% SDS. The membrane was then exposed to a Biomax MS film (Kodak, Rochester, NY, USA).

### 2.4 Transfections and plasmids

Subconfluent cells were transfected with TransIT‐X2 reagent (Mirus, Madison, WI, USA) according to the manufacturer's instructions. At 48 h, cells were seeded in 10‐cm plates with media containing a selection drug (0.5–1 µg·mL^−1^ puromycin, 50 µg·mL^−1^ hygromycin B, and 400 µg·mL^−1^ G418). Cells were transfected with the following plasmids: pGene Clip‐hygromycin negative control (GGAATCTATTCGATGCATAC) (QUIAGEN, Hilden, Germany), referred through the text as V; pGeneClip shMYBBP1A (TCCCTGTCACGCCTACTTTCT) (QIAGEN #3336312 KH08420H), referred through the text as sh; pRetrosuper‐puro, referred through the text as V2; pRetrosuper shMYBBP1A (GCAGAAGGAGTTCAAGAGACTCCTTCTGCAGCTTGTTCTTTTTGGAA), referred through the text as sh2; pRetrosuper scramble negative control (OriGene #TR30012, Rockville, MD, USA), pRetrosuper shPGC1α (TCTGGTACACAAGGCAATAACTCCACCAA) (OriGene #TR310260C), pBABE‐puro, pBABE‐puro‐YFP, pcDNA3‐neomycin, and pcDNA3‐2xFlag‐human‐c‐MYB (kindly provided by Shengao Jin).

### 2.5 Competition assay

Equal number (50% vs 50%) of V‐YFP^+^ cells (control), doubled transfected with pGene Clip‐hygromycin negative control and pBABE‐puro‐YFP vectors, and shMYBBP1A cells, doubled transfected with pGeneClip shMYBBP1A and pBABE‐puro vectors, were seeded in the same dish at complete, low‐glucose, and glutamine‐only media. Percentage of cells was analyzed by FACS the day after seeding. Then, cells were cultured during 15–30 days and the final percentage of cells was counted on an analyzer cytometer (Cedex, France) FACS BD Fortessa.

### 2.6 Growth curve assay and growth in soft agar

We followed a protocol previously described by Guijarro *et al. *(2007) in different media conditions.

### 2.7 Xenografted tumors

Tumorigenicity was assayed by the subcutaneous injection of 5 × 10^6^ cells of 786‐O and 10^6^ of ACHN, A498, and CaKi‐1 cell lines into the right flanks of 4‐week‐old female athymic nude mice. Animals were examined weekly. After 100 or 180 days, depending on cell lines, mice were sacrificed, tumors were extracted and conserved under −80 °C until RNA extraction. All animal experiments were performed according to the experimental protocol approved by the IBIS and HUVR Institutional Animal Care and Use Committee (0309‐N‐15).

### 2.8 Sensitivity to rotenone

In a 96‐well plate, 5 × 10^3^ cells per well were seeded in media with 100 mg·L^−1^ glucose without glutamine or media with glutamine but without glucose. Cells were cultured under normoxic and hypoxic (5%) conditions. After 14–18 h, cells were treated with increasing concentrations of rotenone (0–300 µm) (R8875; Sigma). After 24 h, cell viability was measured with the MTS assay (G3581; Promega, Fitchburg, WI, USA) according to the manufacturer's instructions.

### 2.9 Sensitivity to 2‐deoxy‐d‐glucose

In a 6‐well plate, 10^3^ cells per well were seeded in full media. Cells were treated with 2‐deoxy‐D‐glucose (2DG) (1 mm) (D8375; Sigma‐Aldrich) or water (control). Once colonies were formed, plates were stained with crystal violet, resolubilized in 20% acetic acid, and quantified at 595 nm as a relative measure of colony density.

### 2.10 Mitochondrial ROS measurement

In a 6‐well plate, 1.5 × 10^5^ cells per well were seeded in full media, low‐glucose media, and glutamine‐only media. After 16–18 h, fresh media were added 30 min before beginning the MitoSox protocol. After the media were removed, 1 mL of MitoSox Red (Invitrogen #M36008, Eugene, OR, USA) at 5 µm final concentration was added in each well, and cells were incubated in the dark. Labeled cells were suspended in PBS containing 2% FBS and 5 mm EDTA and analyzed on a FACS Canto II cytometer (Franklin Lakes, NJ, USA).

### 2.11 Q‐RT–PCR

Total RNA from cell lines and xenografted tumors was purified using the ReliaPrep™ RNA Tissue Miniprep System (Promega) according to the manufacturer's instructions. Reverse transcription was performed with 1 μg of mRNA using the High‐Capacity cDNA Reverse Transcription Kit (Life Technologies, Carlsbad, CA, USA), according to the manufacturer’s recommendations. The PCR mixture (10 μL) contained 2 μL of the reverse transcriptase reaction product diluted 1 : 3, 2.5 μL of water, 5 μL of GoTaqR Probe qPCR Master Mix (Promega), and 0.5 μL of the appropriate TaqMan Assay (20×) (Applied Biosystems, Buckinghamshire, UK). We used the following probes: β‐actin (Hs0160665_g1), MYBBP1A (Hs00959671_m1), MYB (Hs00920556_m1), PGC1α (Hs01016719_m1), SGLT1 (Hs01573790_m1), GLUT4 (Hs00168966_m1), HK2 (Hs00606086_m1), PFKM (Hs00175997_m1), GAPDH (Hs03929097_g1), PGK1 (Hs00943178_g1), PGAM1 (Hs01652468_g1), PKM (Hs00761782_g1), and LDHA (Hs01378790_g1).

### 2.12 Protein isolation and western blot analysis

Western blots were performed as previously described elsewhere. Membranes were incubated with the following primary antibodies: anti‐MYBBP1A (Proteintech #14524‐AP, Rosemont, IL, USA ), anti‐PGC1α (Abcam #ab54481, Cambridge, UK), anti‐SGLT1 (Abcam #ab14685), anti‐p38 MAPK (Cell Signaling #9212, Danvers, MA, USA), and anti‐phospho‐p38 MAPK (T180/Y182) (Cell Signaling #9215). α‐Tubulin (Sigma #T9026) was used as a loading control. Horseradish peroxidase‐labeled rabbit anti‐mouse (Abcam #ab 97046) and goat anti‐rabbit (Abcam #ab 97051) secondary antibodies were used. The proteins were detected using an ECL detection system (Amersham Biosciences) and Bio‐Rad ChemiDoc XRST (Berkeley, CA, USA).

### 2.13 Statistical analyses

Statistical analyses of experiments were performed using graphpad prism (La Jolla, CA, USA) (6.01 for Windows). Control samples and MYBBP1A shRNA samples were compared using the unpaired Student’s *t*‐test or Student’s *t*‐test with Welch’s correction, as appropriate. Experiments were performed for a minimum of three times independently and always performed in triplicate samples.

### 2.14 Analysis of cancer patient databases

We used the R2 Genomics analysis and visualization platforms (http://hgserver1.amc.nl). We used six databases, grouped into three sets: EXPO (including several renal tumor subtypes), TCGA (composed of three tumor simple databases: clear cell renal cell carcinoma database (ccRCC), papillary renal cell carcinoma database (pRCC), and chromophobe renal cell carcinoma (chRCC), and Dykema‐Kort (composed of two mixed databases that contain normal and tumor samples). To determine the genes whose expression correlates with *MYBBP1A* expression, we analyzed the following KEGG pathways: ‘glycolysis and gluconeogenesis’ and ‘TCA cycle’. We also added the list of transcription factors that are targets of MYB gene defined in the GeneCards database (http://www.genecards.org) to the R2 platform and analyzed the correlation of the expression of these genes with *MYBBP1A* expression. We selected genes that correlate with *MYBBP1A* expression with a *P*‐value < 0.05. KEGG classification of the overlapped genes was performed with the Enrich platform (http://amp.pharm.mssm.edu/Enrichr/). Finally, heat maps of genes from ‘transcription factors target of MYB gene’ were generated using city‐block distances. Heat maps of TCA cycle genes were generated by Euclidean distance.

## 3 Results

### 3.1 MYBBP1A loss in human tumors

To identify signals with the ability to overcome environmental stress, providing new directions for tumor evolution, we performed a genetic loss‐of‐function screen (Leal *et al.*, 2008). NIH3T3 cells were seeded and infected with a saturated library of antisense fragments. Then, cells were grown in the absence of glucose (Fig. S1A). We identified one antisense fragment that reduced MYBBP1A protein expression by 50% and allowed the cells to grow under these restrictive conditions (Fig. S1B).

However, if the downregulation of MYBBP1A is an important trait required for the evolution of these cells, it must be maintained throughout tumor growth; therefore, we should be able to identify it in human tumors. To this end, we first checked *MYBBP1A* expression in several arrays of paired normal/tumor tissue samples from the same patients. We identified pancreas, liver, and renal tumors as those in which the signal was significantly decreased by at least 50% with respect to normal tissue (Fig. S2). Furthermore, pVHL is frequently lost in renal cancer so that we decided to use renal carcinoma cell lines as physiological models in our study.

### 3.2 MYBBP1A downregulation activates PGC1α

**Figure 1:**
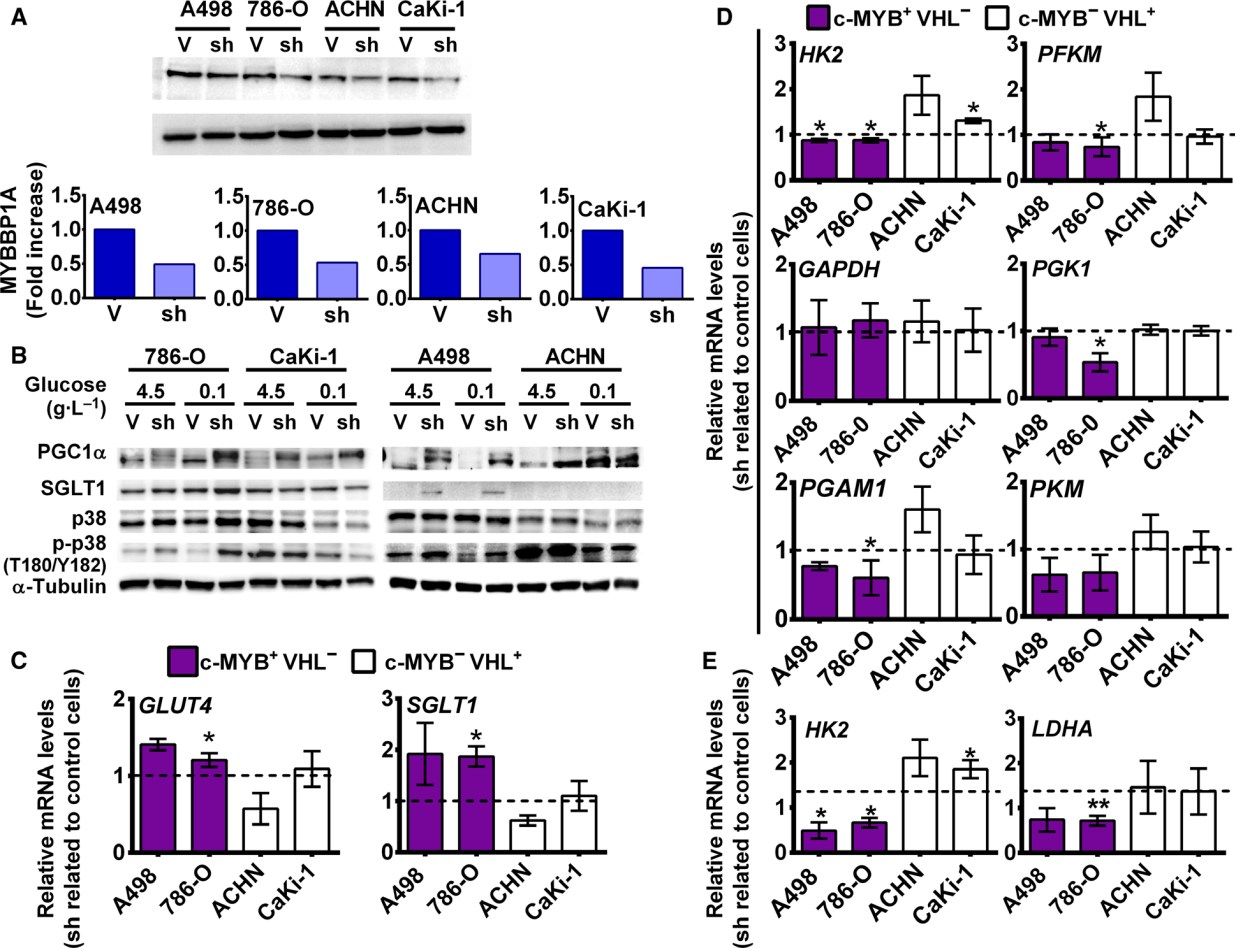
Downregulation of MYBBP1A induces direct activation of PGC1α. (A) Downregulation of MYBBP1A by the expression of a specific shRNA. Cell lines were transfected with *MYBBP1A* shRNA (sh) or a scramble vector (V). After selection, cells were grown to 80% confluence, and proteins were extracted. The figure shows the western blot results of MYBBP1A expression in all cell lines and the quantification of MYBBP1A expression in cells expressing *MYBBP1A* shRNA (sh) related to the scramble vector (V). (B) PGC1α, SGLT1, p38, and p‐p38 (T180/Y182) levels at high‐ (4500 mg·L^−1^) and low‐glucose (100 mg·L^−1^) media were measured by WB. (C) A498, 786‐O, ACHN, and CaKi‐1 cells expressing the scramble vector (V) or MYBBP1A shRNA (sh) were cultured in low‐glucose (100 mg·L^−1^) media**.** *SGLT1* and *GLUT4* mRNA levels were measured by Q‐RT‐PCR. Graphs show mRNA levels of cells with reduced levels of MYBBP1A (sh) related to control cells (V).(D) A498, 786‐O, ACHN, and CaKi‐1 cells expressing the scramble vector (V) or MYBBP1A shRNA (sh) were cultured in low‐glucose (100 mg·L^−1^) media**.** *HK2, PFKM, GAPDH, PGK1, PGAM1,* and *PKM* mRNA levels were measured by Q‐RT‐PCR. Graphs show mRNA levels of cells with reduced levels of MYBBP1A (sh) related to control cells (V). (E) Quantification of *HK2* and *LDHA* levels from xenografted tumors (*N* = 4) by Q‐RT‐PCR. Graphs show mRNA levels of cells with reduced levels of MYBBP1A (sh) related to control cells (V). (C, D) Graphs show the mean ± SD of three independent experiments performed in triplicate. (C–E) Statistical test: unpaired Student’s *t*‐test with Welch’s correction, *P < 0.05; **P < 0.01; ***P < 0.001.

To explore the effect of MYBBP1A downregulation on the four renal carcinoma cell lines selected (Table S1), we silenced *MYBBP1A* with a shRNA and achieved approximately 40–50% reduction of the protein (Fig. 1A). Previous literature data suggest a direct relationship between MYBBP1A and PGC1α since MYBBP1A may bind and repress PGC1α (Fan *et al.*, 2004). This binding is disrupted by p38 phosphorylation of PGC1α (Fan *et al.*, 2004). To study this relationship in our cellular model, we measured the levels of PGC1α, phosphorylated p38, and SGLT1 (a target gene of PGC1α) (Corpe *et al.*, 2001; Yu *et al.*, 2017). We observed an increase in PGC1α levels and an upwards shift of the PGC1α band, probably due to post‐translational phosphorylation that occurs only in A498 and 786‐O cells. We also observed an increase in phosphorylated p38 and SGLT1 levels in these cells (Fig. 1B).

To confirm the activation of PGC1α due to MYBBP1A downregulation, we measured the transcriptional expression of two PGC1α target genes: *SGLT1* and *GLUT4* (Corpe *et al.*, 2001; Michael *et al.*, 2001; Oriente *et al.*, 2008; Yu *et al.*, 2017). We observed an increase in the mRNA levels of both transporters when we reduced the expression of MYBBP1A in cells that express c‐MYB, but this increase was not observed in the c‐MYB‐negative cell lines ACHN and CaKi‐1 (Fig. 1C).

It has been suggested that PGC1α represses glycolysis (Lim *et al.*, 2014), so we wondered whether MYBBP1A downregulation deregulates the expression of glycolytic genes. To confirm this, we measured the mRNA levels of several glycolytic genes and we found that downregulation of MYBBP1A represses *HK2, PFKM, PGAM1,* and *PGK1*, which may decrease glycolysis in 786‐O and A498 cells under low glucose concentration (Fig. 1D). Reduction of *HK2* transcription was reproduced with a second shRNA against *MYBBP1A* (Fig. S3). On the other hand, the expression of these genes was not reduced in c‐MYB‐negative cell lines when MYBBP1A was downregulated (Fig. 1D). This result was also obtained *in vivo*, where we observed reduced expression of *HK2* and *LDHA* in xenografted tumors from 786‐O and A498 cells with downregulated MYBBP1A compared to tumors from control cells. Again, this reduction was not observed in xenografted tumors from ACHN and CaKi‐1 cell lines (Fig. 1E).

**Figure 2:**
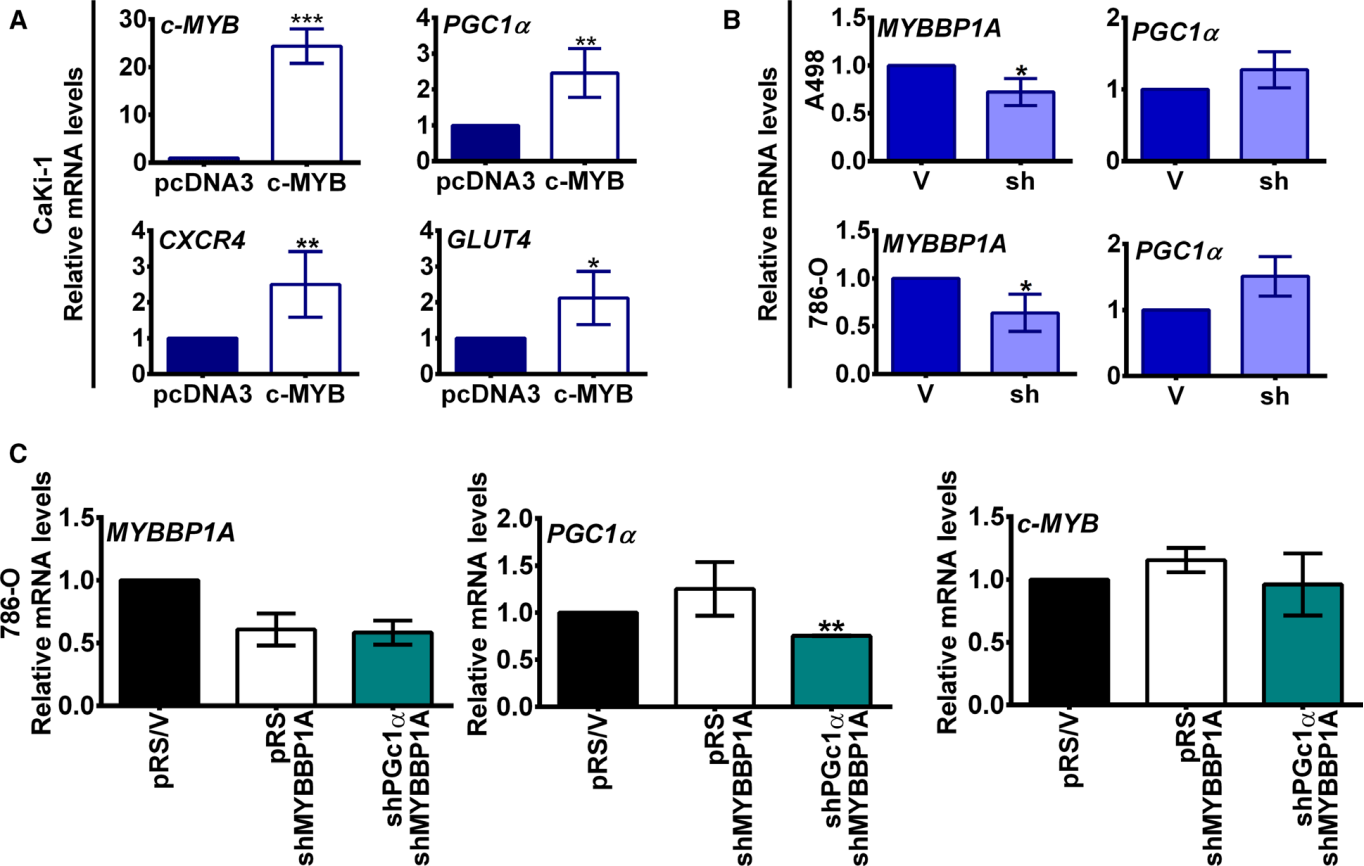
Downregulation of MYBBP1A induces indirect activation of PGC1α through c‐MYB. (A) c‐MYB overexpression induces the transcription of *PGC1α*. The CaKi‐1 cell line was transfected with *c‐MYB* cDNA or the empty vector (pcDNA3). After selection*, c‐MYB, PGC1α, CXCR4,* and *GLUT4* mRNA levels were measured by Q‐RT‐PCR. Graphs show mRNA levels of CaKi‐1 cells expressing *c‐MYB* cDNA related to control cells. (B) Downregulation of MYBBP1A leads to an increase in *PGC1α* mRNA levels. *MYBBP1A* and *PGC1α* mRNA levels of A498 and 786‐O cells expressing the scramble vector (V) or MYBBP1A shRNA (sh) were measured by Q‐RT‐PCR. Graphs show mRNA levels in cells with reduced levels of MYBBP1A (sh) related to control cells (V). (C) 786‐O cells expressing MYBBP1A shRNA were transfected with PGC1α shRNA (shPGC1α/shMYBBP1A) or a scramble vector (pRS/shMYBBP1A). 786‐O control cells were transfected with the scramble vector (V/pRS). After selection, *MYBBP1A*, *PGC1α,* and *c‐MYB* mRNA levels were measured by Q‐RT‐PCR. Graphs represent mRNA levels of each cell line normalized to mRNA levels of control cells (pRS/V). (A–C) Graphs show the mean ± SD of three independent experiments performed in triplicate. Statistical test: unpaired Student’s *t*‐test with Welch’s correction, *P < 0.05; **P < 0.01, ***P < 0.001.

As the downregulation of MYBBP1A induces the activation of PGC1α only in cells with high levels of c‐MYB, we also explored whether c‐MYB may mediate PGC1α activation by MYBBP1A reduction. Thus, we overexpressed c‐MYB in CaKi‐1 cells and measured the expression of *PGC1α*, *GLUT4,* and *CXCR4* (a target gene of c‐MYB controlled by c‐MYB transactivation activity) (Quintana *et al.*, 2011) (Fig. 2A). We observed increases in *PGC1α* and *GLUT4* mRNA levels upon c‐MYB overexpression (Fig. 2A). A similar increase in *PGC1α* was observed in c‐MYB‐positive cells after MYBBP1A was downregulated (Fig. 2B). In addition, we previously reported that A498 MYBBP1A‐downregulated cells metastasized in some host mice before the primary tumors reached large sizes while control cells did not, the *MYBBP1A* levels being lower in metastasis than in primary tumors from xenografted mice (Felipe‐Abrio *et al.*, 2019). We wondered whether *PGC1α* expression pattern would be different in primary tumors versus metastasis, so we measured *PGC1α* mRNA levels and we found an increase in *PGC1α* in metastasis compare with primary tumors from A498‐downregulated cells (Fig. S4).

Alternatively, we downregulated *PGC1α* expression in 786‐O cell line using shRNA to explore whether PGC1α affects *c‐MYB* expression levels. We observed that PGC1α downregulation has no effect on *c‐MYB* mRNA levels (Fig. 2C).

Taken together, our data show that MYBBP1A downregulation induces PGC1α activation and that c‐MYB plays a role in PGC1α modulation induced upon a loss of MYBBP1A. This PGC1α activation suggests the reorientation of tumor cell metabolism toward OXPHOS in MYBBP1A‐downregulated and c‐MYB‐positive cells.

### 3.3 Metabolic plasticity in MYBBP1A‐downregulated cells

**Figure 3:**
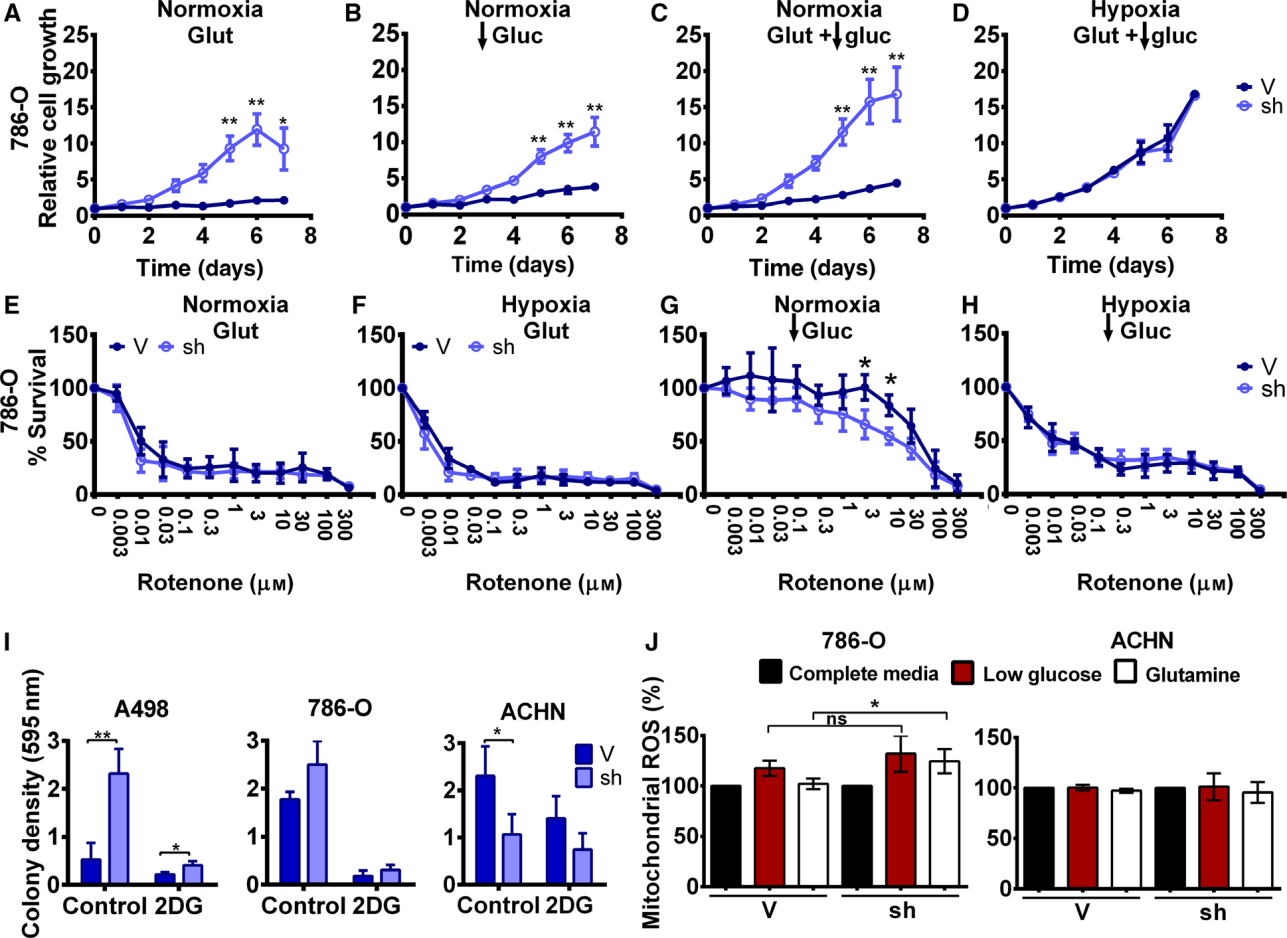
Downregulation of MYBBP1A induces metabolic plasticity in the 786‐O cell line. (A) 786‐O cells expressing the scramble vector (V) or MYBBP1A shRNA (sh) were cultured in glutamine‐only media, low glucose (100 mg·L^−1^) without glutamine media (B), low glucose (100 mg·L^−1^) with glutamine media (C), and low glucose (100 mg·L^−1^) with glutamine media in hypoxic conditions (5%)(D). Cell growth was measured over 7 days. (E–H) Reduction of MYBBP1A in 786‐O cells increases sensitivity to rotenone at low glucose (100 mg·L^−1^) in normoxic conditions. 786‐O cells expressing the scramble vector (V) or MYBBP1A shRNA (sh) were cultured in glutamine‐only media (E), glutamine‐only media under hypoxic conditions (5%) (F), low‐glucose (100 mg·L^−1^) media (G), and low‐glucose (100 mg·L^−1^) media under hypoxic conditions (5%) (H). Cells were treated with increasing rotenone concentrations (0‐300 µm), and the percentage of survival was measured. (I) Clonal growth of control and MYBBP1A‐downregulated cells treated with 2DG (1 mm). Graphs show the relative measure of colony density in control and 2DG treatment conditions. (J) MYBBP1A downregulation increases OXPHOS metabolism in the 786‐O cell line but not in the ACHN cell line. 786‐O and ACHN cells expressing the scramble vector (V) or MYBBP1A shRNA (sh) were cultured in complete full media, low‐glucose media (100 mg·L^−1^), or glutamine‐only media. Mitochondrial ROS production was measured by MitoSOX staining and flow cytometry analysis. Graphs represent the mitochondrial ROS of control cells, and cells with reduced levels of MYBBP1A in low‐glucose or glutamine‐only media related to complete full media as a percentage. Glut, glutamine; gluc, glucose. (A–J) Graphs show the mean ± SD of three independent experiments performed in triplicate. Statistical test: unpaired Student’s *t*‐test, *P < 0.05; **P < 0.01.

Metabolic plasticity relies on the ability of cells to rewire the existing metabolic pathways depending on cellular needs and nutrient availability. One of the most important metabolic adaptations in absence of glucose is to use glutamine for energy production and anabolic reactions, which enhances OXPHOS metabolism. During glutaminolysis, glutamine is converted into α‐ketoglutarate, which enters directly into the Krebs cycle. To measure the metabolic plasticity of cells with reduced expression of MYBBP1A, we cultured the 786‐O cell line in different conditions of nutrient limitation. Tumor cells expressing the control vector do not grow in medium containing only glutamine (Fig. 3A); however, cells with low levels of MYBBP1A grow fast (Fig. 3A), indicating a switch to OXPHOS metabolism. Similar effects can be observed when the cells were grown under very low glucose concentrations (Fig. 3B). The effect is even more obvious when the cells were grown under glutamine and low glucose concentrations (Fig. 3C). In this media, control cells showed initial growth as consequence of the low glucose. However, the growth of cells with low MYBBP1A is twofold to fourfold faster (Fig. 3C). Finally, during hypoxia, the advantage of having oxygen disappears for cells using OXPHOS, and both cell strains grow equally regardless of the ATP source (Fig. 3D).

The complex I of the mitochondrial electron transport chain is used to produce ATP in OXPHOS metabolism (Lorendeau *et al.*, 2016). Rotenone inhibits this complex and is very poisonous to cells, but interestingly, it is identically toxic for both control cells and cells with low MYBBP1A levels that are growing in the presence (Fig. 3E) or absence (Fig. 3F) of oxygen in media only containing glutamine. However, for cells growing under low glucose conditions, which allow some plasticity and reorientation to cells growing in the presence of oxygen, rotenone is significantly more toxic in cells with reduced levels of MYBBP1A, while control cells are more resistant to this poison (Fig. 3G). However, under hypoxic conditions, this difference disappears because the lack of oxygen prevents metabolism reorientation (Fig. 3H). Similar data were obtained using a second shRNA against *MYBBP1A* (Fig. S5).

Alternatively, we tested the sensitivity of A498, 786‐O, and ACHN cells to 2DG, a well‐known inhibitor of glycolysis. We seeded the cells at low density and cultured them in media with or without 2‐DG. A498‐ and 786‐O MYBBP1A‐downregulated cells formed more colonies than control cells in both control and 2DG treatment conditions (Fig. 3I, Fig. S6), pointing to an increased resistance to 2DG treatment. However, MYBBP1A downregulation in the ACHN cell line reduced the number of colonies in both conditions (Fig. 3I, Fig. S6). The increased resistance to 2DG of c‐MYB‐positive cells also suggests a switch to oxidative metabolism.

To explore whether MYBBP1A downregulation increases metabolic plasticity in different molecular contexts, we performed surrogated tumorigenic assays with A498, ACHN, and CaKi‐1 cell lines in low‐glucose media. We found that A498 cells with reduced levels of MYBBP1A grew faster than cells expressing only the empty vector (Fig. S7A). However, ACHN and CaKi‐1 grew at the same rate in both the control cells and the cells with MYBBP1A downregulation (Fig. S7A). Similar data were obtained in a soft agar assay; A498 cell lines with reduced levels of MYBBP1A formed more colonies than cells expressing scramble vector (Fig. S7B). However, ACHN and CaKi‐1 with reduced MYBBP1A formed the same number of colonies or an even lower number than the control cells (Fig. S7B).

We also confirmed the reorientation toward OXPHOS by measuring mitochondrial ROS, as mitochondrial superoxide is generated as a by‐product of oxidative phosphorylation. To this end, we cultured control and MYBBP1A‐downregulated 786‐O and ACHN cells in either high‐ or low‐glucose media or glutamine‐only media, and we measured mitochondrial ROS by flow cytometry. We observed an increase in mitochondrial ROS in 786‐O cells with reduced MYBBP1A when the cells were cultured in glutamine‐only media, but there was no difference in the ACHN cell line (Fig. 3J).

Finally, since our aim was to discover genetic events that give competitive advantage in harsh microenvironments, we performed competition assays. We cocultured control cells (labeled with YFP, V‐YFP+) and equal number cells with reduced MYBBP1A (with empty vector, shMYBBP1A) in the same dish and with different media conditions and let the mix population evolve. At day 1, equal number of V‐YFP^+ ^(control) and shMYBBP1A (50% vs 50%) were seeded in the same dish (Table 1). We observed that the percentage of each subpopulation barely changed after coculture the three cell lines tested (786‐O, ACHN, and A498) at high glucose condition. However, at low‐glucose or in glutamine‐only media, the percentage of control V‐YFP^+^ cells at the end of the experiment was greatly reduced in 786‐O and A498 cells, indicating that the cells with low MYBBP1A were more competitive and grew better overcoming the control population in these restrictive conditions. However, in ACHN cells, we observed an increase in the control subpopulation, indicating that in this cell line the reduction of MYBBP1A did not provide a selective advantage in these restrictive media (Table 1).

**Table 1:** Coculture experiments in different media.

Coculture	Glucose 4500 mg·L^−1^		Glucose 100 mg·L^−1^		Glutamine	
	% V‐YFP^+^ cells		% V‐YFP^+^ cells		% V‐YFP^+^ cells	
786‐O V‐YFP vs 786‐O shMYBBP1A	**Day 1**	**Day 15**	**Day 1**	**Day 15**	**Day 1**	**Day 15**
	50	52.8	50	17.5	50	21.2
A498 V‐YFP vs A498 shMYBBP1A	**Day 1**	**Day 30**	**Day 1**	**Day 30**	**Day 1**	**Day 30**
	50	59.7	50	9.4	50	10
ACHN V‐YFP vs ACHN shMYBBP1A	**Day 1**	**Day 15**	**Day 1**	**Day 15**	**Day 1**	**Day 15**
	50	63.6	50	69.1	50	70.6

Percentages of V‐YFP^+^ cells, expressing scramble vector and YFP‐vector, at the start (Day 1) and end of the experiment (Day 15/Day 30). Average of three independent experiments.

Taken together, our data indicate that cells with downregulated MYBBP1A have a higher metabolic plasticity regarding glucose limitation, being able to rewire their metabolism to OXPHOS. Interestingly, the effect of MYBBP1A is observed in cells with c‐MYB, which regulates PGC1α.

### 3.4 *MYBBP1A* expression in renal cancer and its role in tumor progression

**Figure 4:**
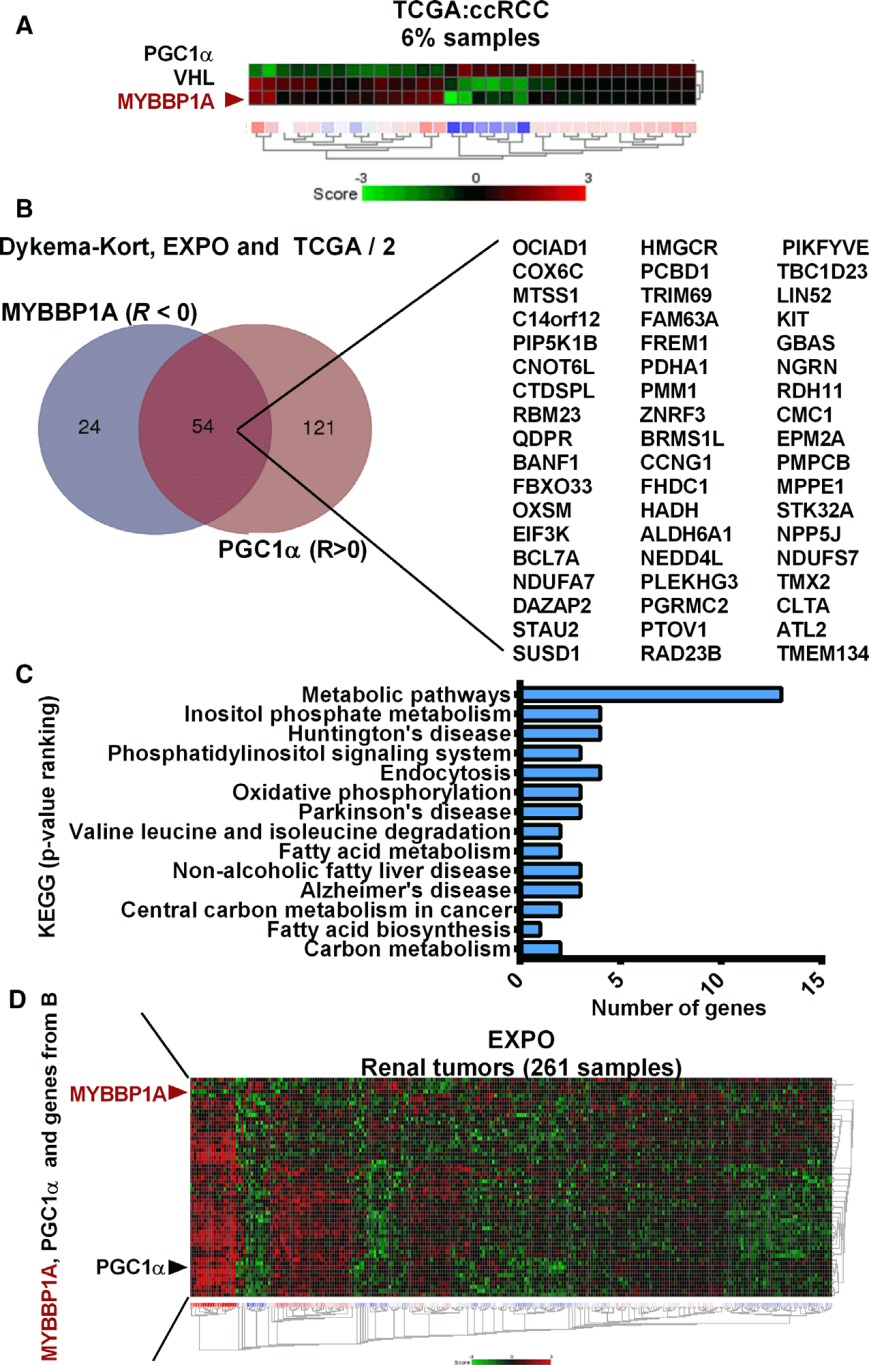
MYBBP1A expression in renal cancer and its correlation with genes involved in signaling pathways in cancer. (A) Heat map of *PGC1α, VHL,* and *MYBBP1A* expression in the TCGA (ccRCC) database. The heat map shows the 3% of samples with lowest *MYBBP1A* and the 3% of samples with highest *MYBBP1A* expression. (B) Venn diagram of c‐MYB target genes that correlate negatively with *MYBBP1A* and positively with *PGC1α* expression with a *P*‐value < 0.05 in at least two of the three databases analyzed. (C) Graph shows the classification by KEGG of c‐MYB target genes that correlate negatively with *MYBBP1A* and positively with *PGC1α* expression with a *P*‐value < 0.05 in at least two of the three databases analyzed. KEGG pathways are ranked by *P*‐value, the metabolic pathways being the KEGG, which shows the lowest *P*‐value. (D) Heat map of *MYBBP1A, PGC1α* and c‐MYB target genes from B in the EXPO database. Heat map was sorted by city‐block distances.

To examine the role of *MYBBP1A* in human tumor progression, in relation to an increase in c‐MYB and PGC1α, we used public transcriptome databases. First, we analyzed the expression *of MYBBP1A, VHL, PGC1α,* and *MYB* genes, functionally related in our cellular model. We focused on the samples with highest and lowest expression levels of *MYBBP1A* (approximately 6% of the samples) and observed that samples with low levels of *MYBBP1A* show low levels of *VHL* and high levels of *PGC1α*. In contrast, samples with high expression levels of *MYBBP1A* show high levels of *VHL* and low levels of *PGC1α* (Fig. 4A).

To determine whether there is a relationship between *MYBBP1A, c‐MYB,* and *PGC1α* in human renal tumors*,* we studied c‐MYB target genes that had a negative correlation with *MYBBP1A* and a positive correlation with *PGC1α* expression with a *P*‐value < 0.05 in at least two of the three databases analyzed. We obtained a list of 54 genes involved in various pathways (Fig. 4B). When we classify these genes by KEGG pathway analysis, we observed that most of the genes belong to metabolic pathways (Fig. 4C). Moreover, the heat map generated using the EXPO database with these 54 genes showed that approximately 8% of samples have low *MYBBP1A* expression, high *PGC1α* expression, and high expression of these 54 genes, suggesting a metabolic shift in these tumor samples (Fig. 4D). Interestingly, the predominant subtype of RCC within this 8% of samples was chRCC, followed by ccRCC (Fig. S8A). In the KORT database, around 32% of samples showed low *MYBBP1A* expression, high *PGC1α* expression, and high expression of c‐MYB target genes, all of these samples being chRCCs or renal oncocytomas (Fig. S8B,C).

**Figure 5:**
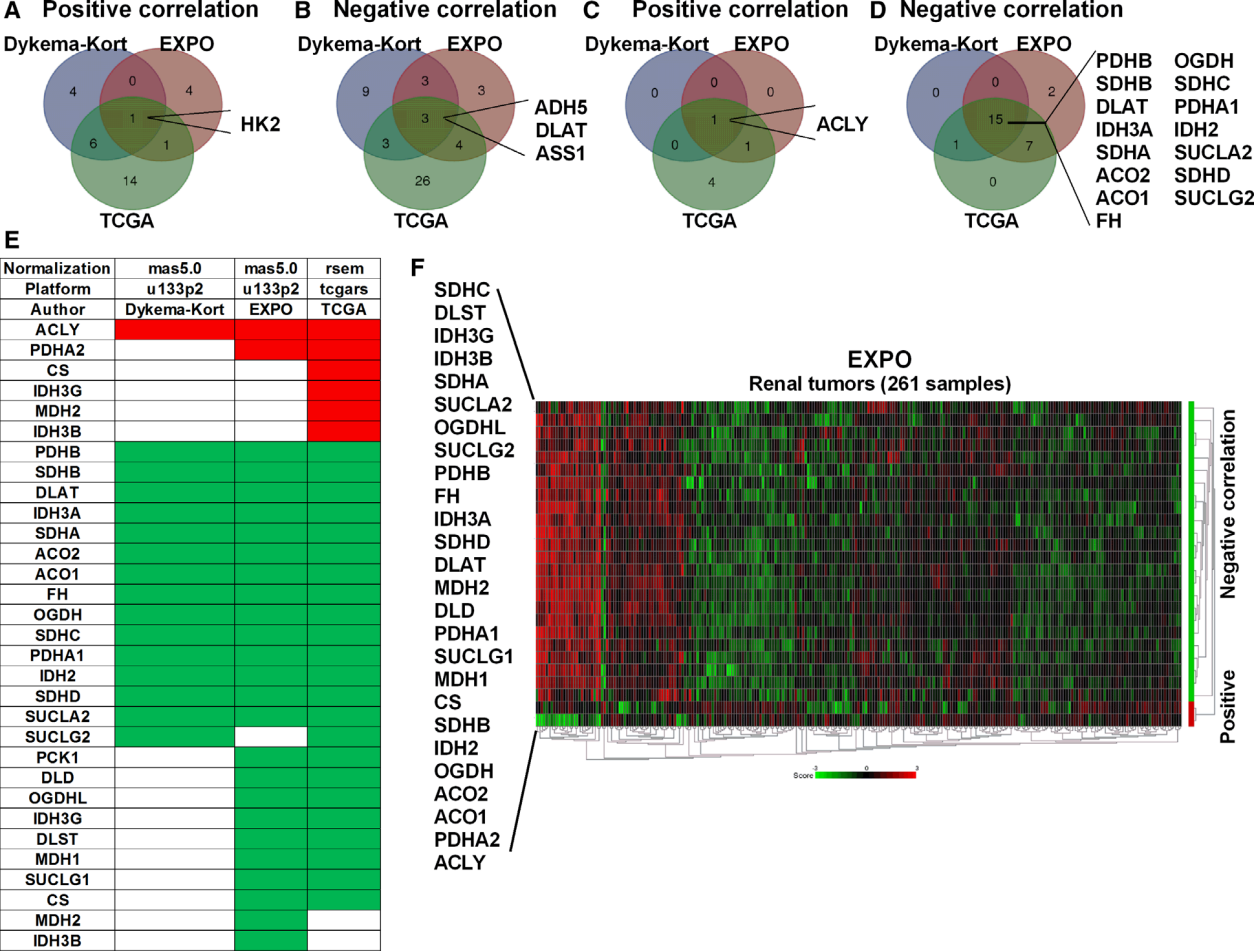
Low *MYBBP1A* expression is associated with a metabolic shift from glycolysis to OXPHOS. (A) Venn diagram of glycolytic genes that correlate positively with *MYBBP1A* expression in the three databases analyzed. (B) Venn diagram of glycolytic genes that correlate negatively with *MYBBP1A* expression in the three databases analyzed. (C) Venn diagram of TCA cycle genes that correlate positively with *MYBBP1A* expression in the three databases analyzed. (D) Venn diagram of TCA cycle genes that correlate negatively with *MYBBP1A* expression in the three databases analyzed. (E) List of TCA cycle genes that correlate positively (red) and negatively (green) with *MYBBP1A* expression in each database. (F) Heat map of the TCA cycle genes that correlate positively (red bar) and negatively (green bar) with *MYBBP1A* in the EXPO database. Heat map was sorted by Euclidean distance

Finally, because we observed a shift from glycolysis to OXPHOS when we reduced *MYBBP1A* mRNA levels in our cellular model, we used these public databases to confirm that this metabolic shift is also observed in tumor samples. We observed a positive correlation between *MYBBP1A* and *HK2*, a key regulatory enzyme in the glycolysis pathway, in all the tested databases (Fig. 5A). Moreover, we observed a negative correlation between *MYBBP1A* and *ADH5, DLAT* and *ACSS1* in the three databases. DLAT and ACSS1 are involved in the production of acetyl‐CoA so that it can be used for oxidation in the TCA (Fig. 5B). Alternatively, we studied the correlation between *MYBBP1A* and genes of the TCA, detecting a positive correlation between *MYBBP1A* and *ACLY* and a negative correlation with 15 other genes involved in the production and oxidation of acetyl‐CoA by TCA (Fig. 5C–E, Table S2). In addition, when we represented the correlation between *MYBBP1A* and genes of the TCA cycle on a heat map, we observed that 9% of the samples have high expression of the genes that showed negative correlation with *MYBBP1A*, suggesting that these samples rely on OXPHOS metabolism (Fig. 5F). Yet again, the majority of these samples were chRCCs, followed by ccRCCs and pRCCS (Fig. S8D). In the KORT database, around 32% of samples showed low *MYBBP1A* expression and high expression of TCA genes, all of these samples being chRCCs or renal oncocytomas (Fig. S8E,F).

These correlations confirm the capability of MYBBP1A to regulate glucose metabolism through c‐MYB and PGC1α in human tumors.

## 4 Discussion

Low glucose levels or the depletion of other nutrients cause tumors to suffer by the conflict between low energetic conditions and the high metabolic requirements. Events that allow tumor cells to grow or resist under these stringent conditions provide selective advantages over the remaining tumor cells directing clonal evolution. By performing a genetic loss‐of‐function screen, we found that downregulation of MYBBP1A allows cells to survive and proliferate under these conditions by switching to OXPHOS metabolism. These phenotypes are regulated through c‐MYB and PGC1α. MYBBP1A is a potent tumor suppressor repressing c‐MYB. Its downregulation derepresses c‐MYB, which leads to transcriptional activation of PGC1α. Therefore, MYBBP1A downregulation indirectly activates PGC1α, switching the metabolism to OXPHOS and generating more energy under low glucose conditions. We believe that the combined effects elicited by MYBBP1A downregulation confer selective advantages over other tumor cells. Moreover, cells with MYBBP1A downregulation can be observed in clinically relevant renal carcinoma tumors, confirming their biological relevance.

MYBBP1A was first identified by its ability to interact specifically with c‐MYB NRD through leucine zipper‐like motifs (Favier and Gonda, 1994; Tavner *et al.*, 1998). Thus, it was suggested that MYBBP1A may modulate c‐MYB activity upon binding to the c‐MYB NRD. Later, it was reported that MYBBP1A also binds and represses PGC1α (Fan *et al.*, 2004). We have found that MYBBP1A reduction results in an increase in c‐MYB activity, which leads to the increment of *PGC1α* expression. Hence, loss of MYBBP1A may simultaneously activate PGC1α by alleviating direct repression (Fan *et al.*, 2004) and increasing the *PGC1α* mRNA and protein levels through c‐MYB. Furthermore, p38 has been described as a PGC1α activator that blocks MYBBP1A binding to PGC1α (Fan *et al.*, 2004). p38 activation is related to the CXCR4 pathway (Broussas *et al.*, 2016; Zhan *et al.*, 2016; Zuo *et al.*, 2016), and since *CXCR4* is a target gene of c‐MYB (Quintana *et al.*, 2011), activation of this transcription factor by loss of MYBBP1A (Felipe‐Abrio *et al.*, 2019) may lead to indirect activation of p38, therefore potentiating a loop leading to PGC1α activation.

PGC1α acts as a stress sensor for tumor cells activated by nutrient deficiency, oxidative damage, and chemotherapy. By participating in the maintenance of metabolic homeostasis, PGC1α promotes tumor cell survival and metastasis in restrictive microenvironments (Tan *et al.*, 2016). Activation of PGC1α switches glycolysis to OXPHOS by decreasing glycolytic gene transcription, derepressing mitochondrial respiration, and increasing mitochondrial biogenesis as well as increasing the expression of glucose transporters, such as GLUT4 (Gannon *et al.*, 2015). We found that loss of MYBBP1A release *PGC1α* activity and transcription activation through c‐MYB switching tumor bioenergetics, increasing glutaminolysis and sensitivity to oxidative channel inhibition. The metabolic switch caused by MYBBP1A downregulation through both c‐MYB and PGC1α activation is translated to increased proliferation in low‐glucose microenvironments, providing cells a competitive advantage to survive in restrictive microenvironments and confirming the tumor suppressor activity of MYBBP1A. In addition, by participating in the maintenance of metabolic homeostasis, PGC1α promotes the survival of tumor cells and metastasis in restrictive microenvironments (Tan *et al.*, 2016). The data from our xenograft models revealed an increment in *PGC1α* expression in metastasis compared to primary tumors, suggesting that PGC1α promotes metastasis as well, but the number of mice where we observed metastasis was low, so further studies will be needed to corroborate this association in mice.

On the other hand, HIF represses PGC1α and CPT1A (Du *et al.*, 2017; LaGory *et al.*, 2015; Soro‐Arnaiz *et al.*, 2016). Perhaps the group of renal carcinoma with low MYBBP1A better counteracts this antioxidative pressure executed by the HIF factors on PGC1α and give some ccRCC with more mitochondrial activity that possibly alters its tumor phenotype. This will also explain the competitive advantage of these low MYBBP1A tumors in a microenvironment with high oxygen and low glucose.

Both presence of c‐MYB and absence of pVHL are required to detect the effect of MYBBP1A downregulation due to the exclusive relationship that we have observed between c‐MYB and pVHL. Furthermore, it has been reported that all MYB family proteins, c‐MYB included, may be able to interact with pVHL, namely with the isoform 3 of pVHL (Okumura *et al.*, 2016). Indeed, B‐Myb is a substrate of pVHL, the interaction being stronger between B‐Myb and isoform 3 of pVHL (Okumura *et al.*, 2016). These suggest that pVHL absence is needed in order to get a relevant expression of c‐MYB, which is a key factor involved in MYBBP1A downregulation response.

Our analysis of public transcriptome databases reveals that the reduction of MYBBP1A expression occurs in a significant percentage of renal tumor samples (approx 9%). We have identified a group of tumor samples that shows low expression of *MYBBP1A* and high expression of *PGC1α* and c‐MYB target genes involved in metabolic pathways. This group of tumor samples also exhibit high expression of genes involved in oxidative metabolism. This finding, together with the increased sensitivity to oxidative channel inhibition caused by MYBBP1A downregulation *in vitro*, suggests a possible alternative therapy for a subgroup of renal carcinoma patients. In fact, tumor cells with low MYBBP1A are more sensitive to mitochondrial respiratory channel inhibition by rotenone, suggesting some degree of dependency of these cells from the oxidative metabolism. Interestingly, the analysis of *MYBBP1A* low tumor samples by different subtypes of RCC points to *MYBBP1A* loss is more frequent in chRCCs, which could be a suitable subgroup to target oxidative pathways as an alternative therapeutic approach.

## 5 Conclusions

Our work reveals that MYBBP1A downregulation simultaneously activates PGC1α directly by alleviating direct repression and indirectly by increasing the *PGC1α* levels through c‐MYB. By PGC1α activation, cellular metabolism is switched from glycolysis to OXPHOS, increasing glutaminolysis and allowing the tumor cells to adapt to harsh microenvironments, which provides a competitive advantage over other tumor cells. Interestingly, cancer cells that express c‐MYB and do not express pVHL are the ones that can take advantage of MYBBP1A downregulation. In conclusion, loss of MYBBP1A is observed in a significant percentage of patients with renal cell carcinoma, who may benefit from cancer therapies that target metabolic pathways.

## Conflict of interest

The authors declare no conflict of interest.

## Author contributions

BF‐A and AC designed the experiments. BF‐A and EMV‐S performed the experiments. AC wrote the manuscript, and AC and BF‐A edited the manuscript. All authors revised the content of the manuscript.

## Supporting information


**Fig. S1** **.** Identification of an antisense fragment against *MYBBP1A* using a genetic loss‐of‐function screen in the absence of glucose.
**Fig. S2** **.** Reduction of *MYBBP1A* expression in renal, pancreas and liver tumors.
**Fig. S3** **.** Downregulation of MYBBP1A with a second shRNA.
**Fig. S4** **.** Expression of *PGC1α* in primary tumors and metastasis.
**Fig. S5** **.** Downregulation of MYBBP1A induces metabolic plasticity in 786‐O cell line with a second shRNA.
**Fig. S6** **.** Clonal growth of control and MYBBP1A downregulated cells treated with 2DG (1mM).
**Fig. S7** **.** MYBBP1A reduction increases tumorigenic properties in c‐MYB+ and pVHL‐ cell lines under low glucose concentrations.
**Fig. S8** **.** Analysis of *MYBBP1A* expression and its correlation with genes involved in metabolic pathways by different subtypes of RCCs.
**Table S1** **.** Characteristics of cell lines.
**Table S2** **.** Correlation between the expression of *MYBBP1A* and genes of the TCA cycle.
