# Supplementary material for: c‐MYB‐ and PGC1a‐dependent metabolic switch induced by MYBBP1A loss in renal cancer
Source: Mol Oncol. 2019 Jun 11;13(7):1519–33. doi: 10.1002/1878-0261.12499 (PMC6599841; doi:10.1002/1878-0261.12499)

**SUPPLEMENTARY INFORMATION**

**c-MYB- and PGC1a-dependent metabolic switch induced by MYBBP1A loss in renal cancer**

**Authors:** Blanca Felipe-Abrio; Eva M. Verdugo-Sivianes and Amancio Carnero *

Supplementary Figure 1. Identification of an antisense fragment against *MYBBP1A* using a genetic loss-of-function screen in the absence of glucose.

Supplementary Figure 2. Reduction of *MYBBP1A* expression in renal, pancreas and liver tumors.

Supplementary Figure 3. Downregulation of MYBBP1A with a second shRNA.

Supplementary Figure 4. Expression of *PGC1α* in primary tumors and metastasis.

Supplementary Figure 5. Downregulation of MYBBP1A induces metabolic plasticity in 786-O cell line with a second shRNA.

Supplementary Figure 6. Clonal growth of control and MYBBP1A downregulated cells treated with 2DG (1mM).

Supplementary Figure 7. MYBBP1A reduction increases tumorigenic properties in c-MYB+ and pVHL- cell lines under low glucose concentrations.

Supplementary Figure 8. Analysis of *MYBBP1A* expression and its correlation with genes involved in metabolic pathways by different subtypes of RCCs.

Supplementary Table 1. Characteristics of cell lines.

Supplementary Table 2. Correlation between the expression of *MYBBP1A* and genes of the TCA cycle.


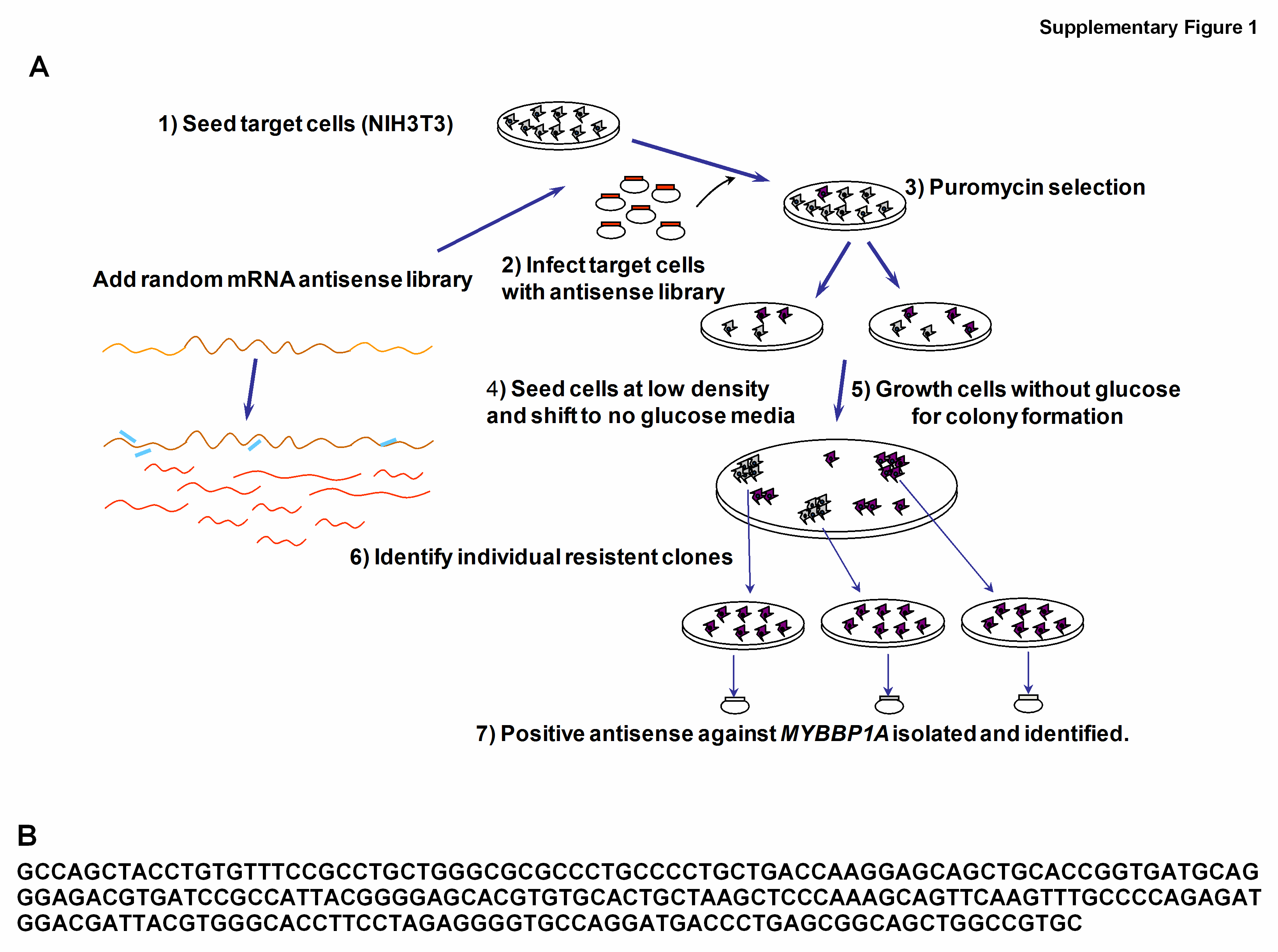


**Supplementary Figure 1. Identification of an antisense fragment against *MYBBP1A* using a genetic loss-of-function screen in the absence of glucose.** (A) Schematic representation of the genetic loss-of-function screen in absence of glucose. (B) Sequence of the antisense fragment against *MYBBP1A*.


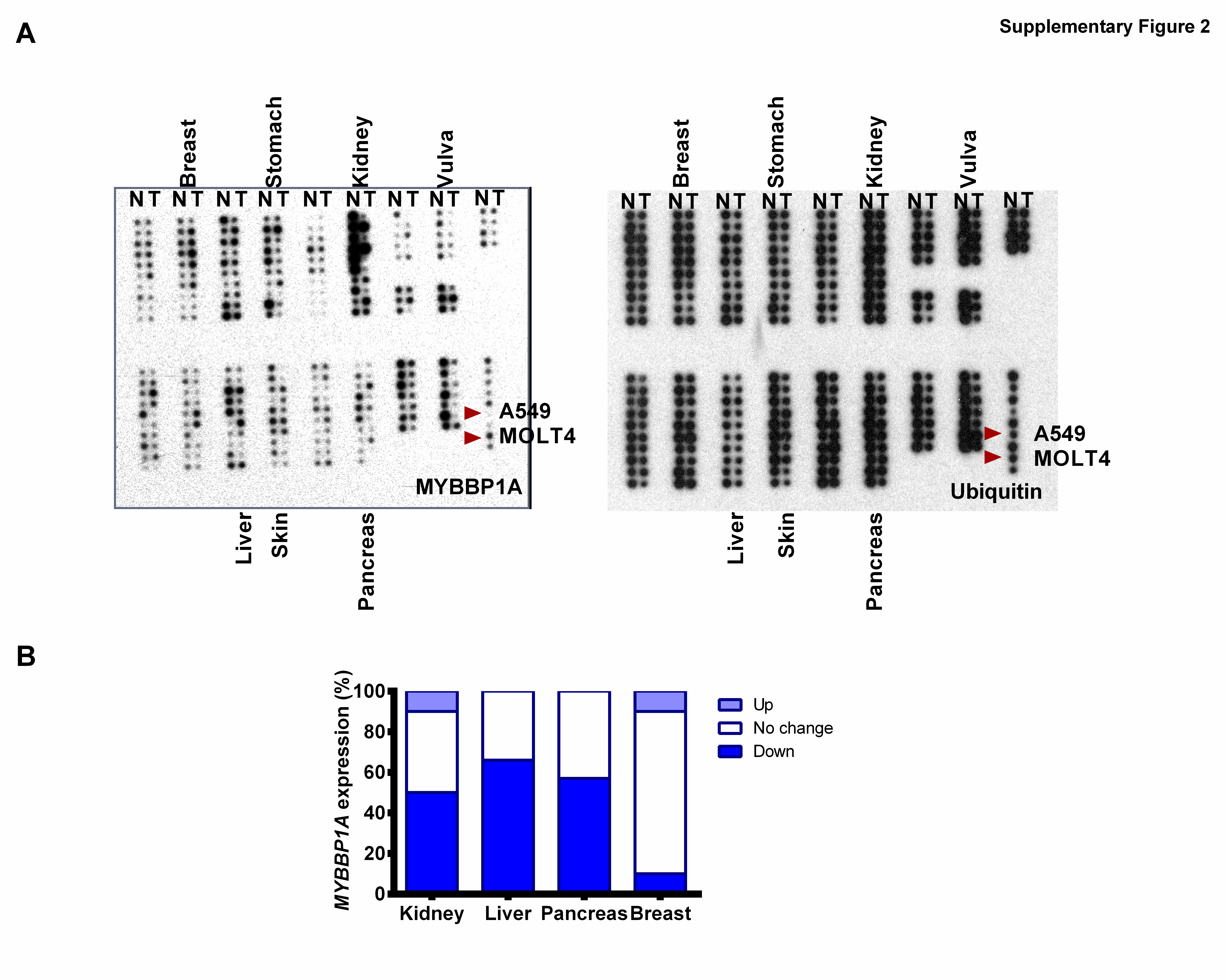


**Supplementary Figure 2. Reduction of *MYBBP1A* expression in renal, pancreas and liver tumors.** (A) Array of paired normal (N)/ tumor (T) RNA samples. cDNA probes of *MYBBP1A* gene were radioactively labeled and hybridized with the array of normal/tumor RNA samples. As an expression control, we hybridized the arrays with an ubiquitin-specific probe. Arrows point human cell lines samples used as controls. We normalized the signals of the probes against the ubiquitin signal and quantified the signal in normal and tumor samples. (B) Percentage of tumor samples with a reduction, increase or no change in *MYBBP1A* expression after normalization.


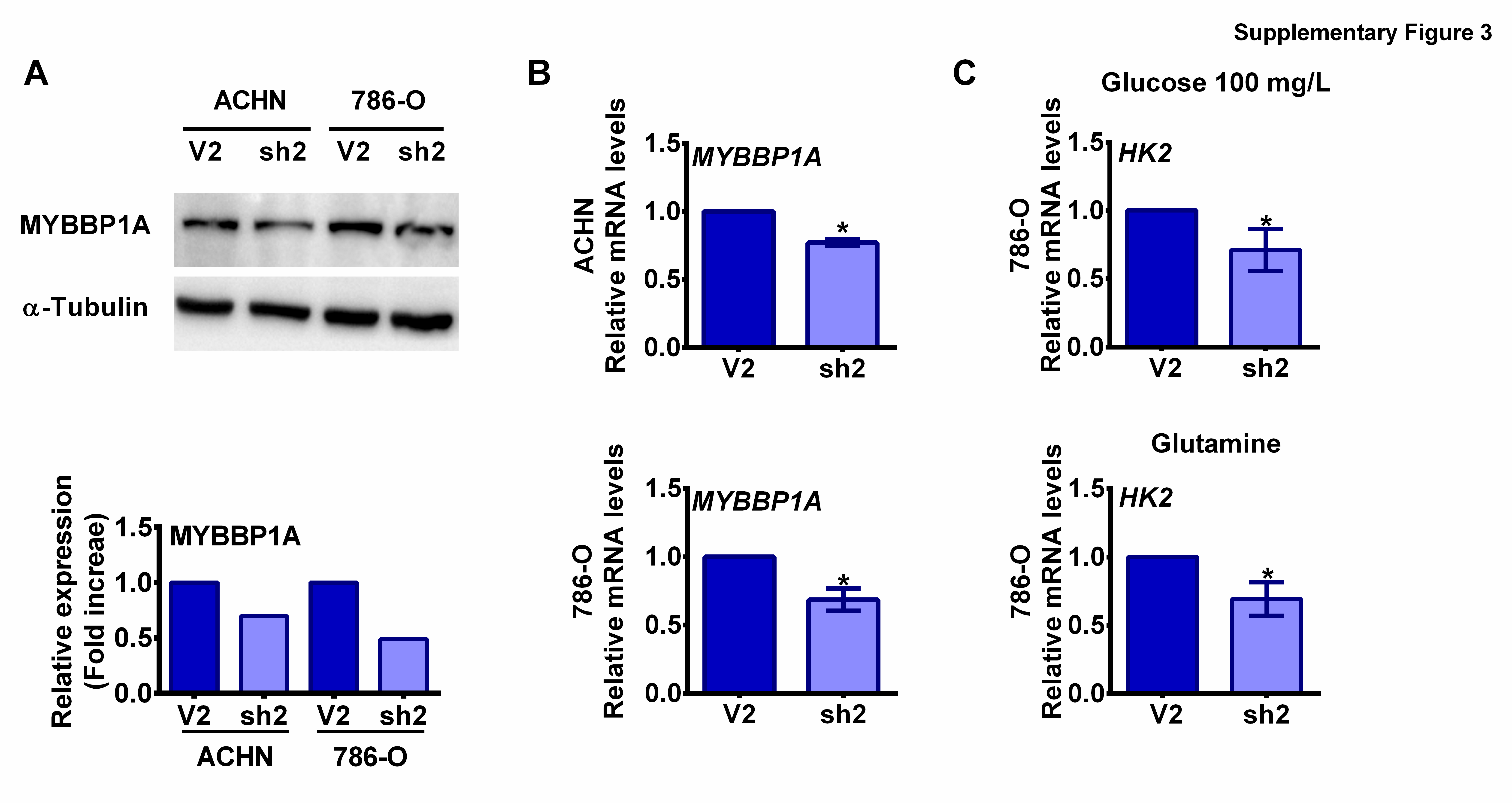


**Supplementary Figure 3. Downregulation of MYBBP1A with a second shRNA.** 786-O cell line was transfected with MYBBP1A shRNA2 (sh2) and an empty vector (V2). After selection, proteins and RNA were extracted when cells reached 80% confluence. MYBBP1A levels were measured by WB (A) and Q-RT-PCR (B). (C) Quantification of *HK2* mRNA levels from 786-O cells expressing MYBBP1A shRNA2 (sh2) or the empty vector (V2) cultured in low glucose (100 mg/L) media (top) and glutamine only media (bottom). Graphs show mRNA levels of cells with reduced levels of MYBBP1A (sh2) related to control cells (V2). *p<0.05.


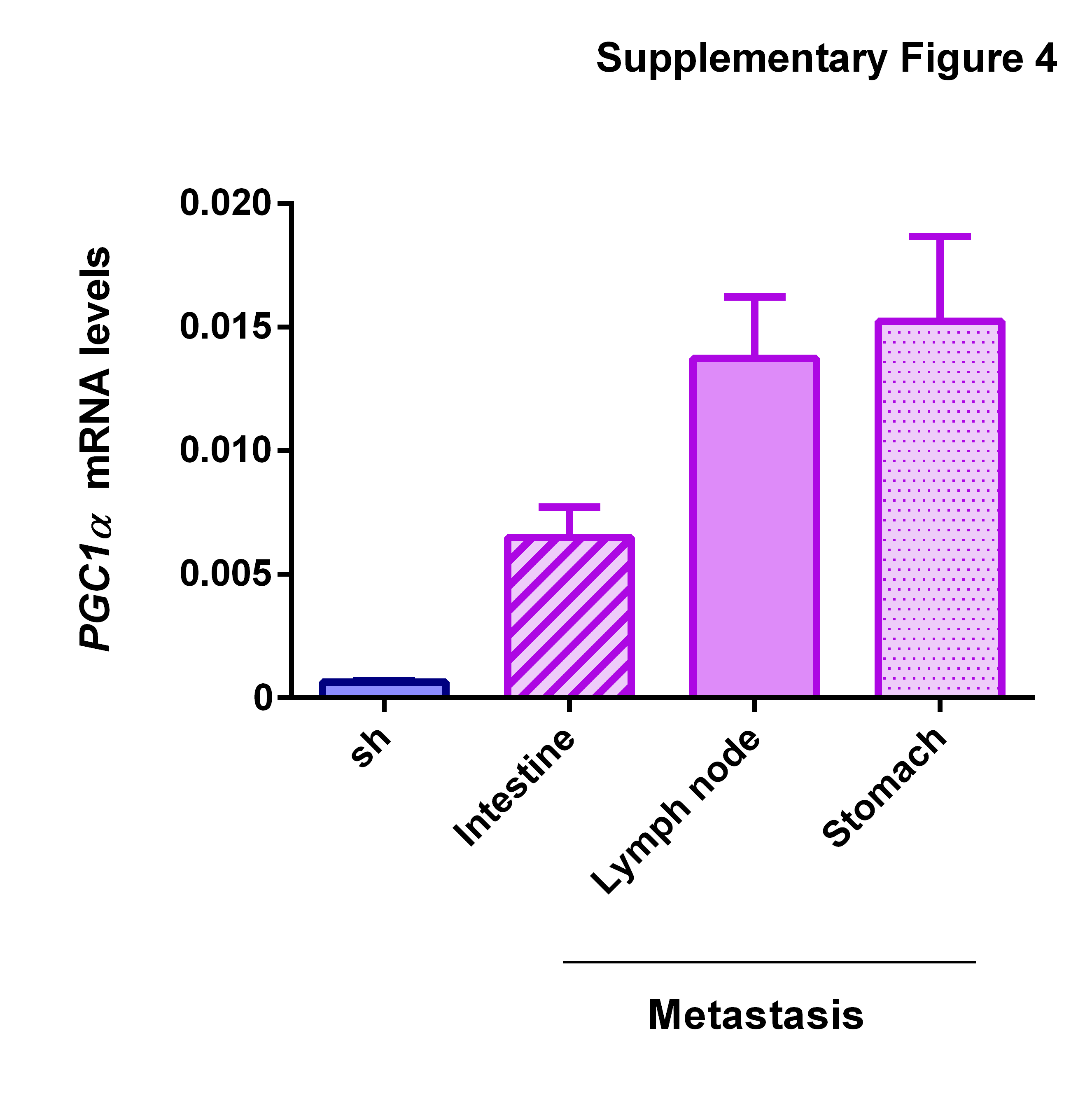


**Supplementary Figure 4. Expression of *PGC1α* in primary tumors and metastasis.** Measurement of *PGC1α* mRNA levels in primary tumors and metastasis from A498 MYBBP1A downregulated cells by Q-RT-PCR.


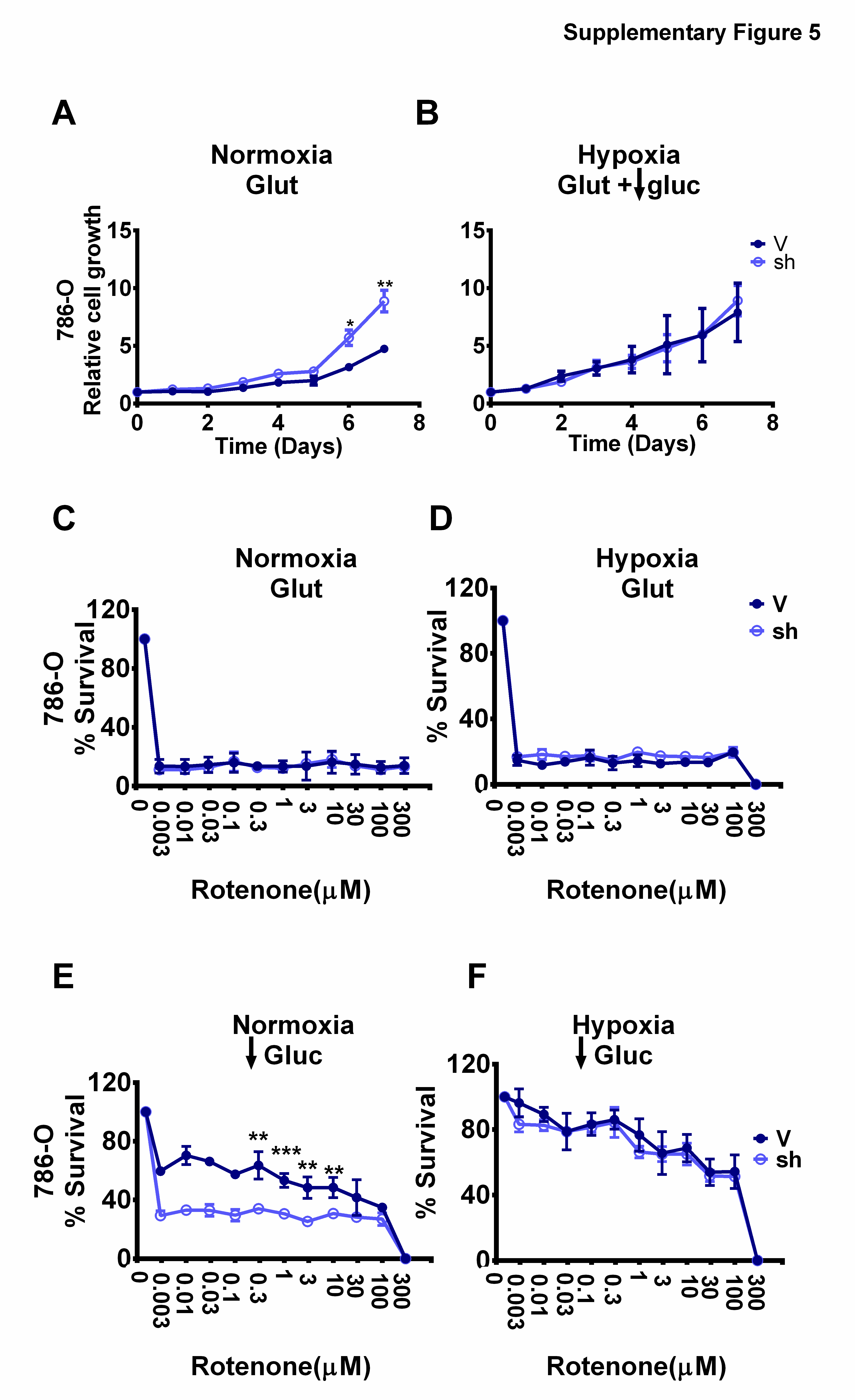


**Supplementary Figure 5. Downregulation of MYBBP1A induces metabolic plasticity in 786-O cell line with a second shRNA.** (A-B) 786-O cells expressing MYBBP1A shRNA2 (sh2) or the empty vector (V2) were cultured in glutamine only media (A) and low glucose (100 mg/L) media with glutamine in hypoxia (5%) (B). Cell growth was measured over 7 days. (C-F) 786-O cells expressing MYBBP1A shRNA2 (sh2) or the empty vector (V2) were cultured in glutamine only media (C), glutamine only media under hypoxic conditions (5%) (D), low glucose (100 mg/L) media (E) and low glucose (100 mg/L) media under hypoxic conditions (5%) (F). Cells were treated with increasing rotenone concentrations (0-300 µM), and the percentage of survival was measured. Graphs show the mean±SD of three independent experiments performed in triplicate. Glut= glutamine; Gluc= glucose; *p<0.05; **p<0.01; ***p<0.001.


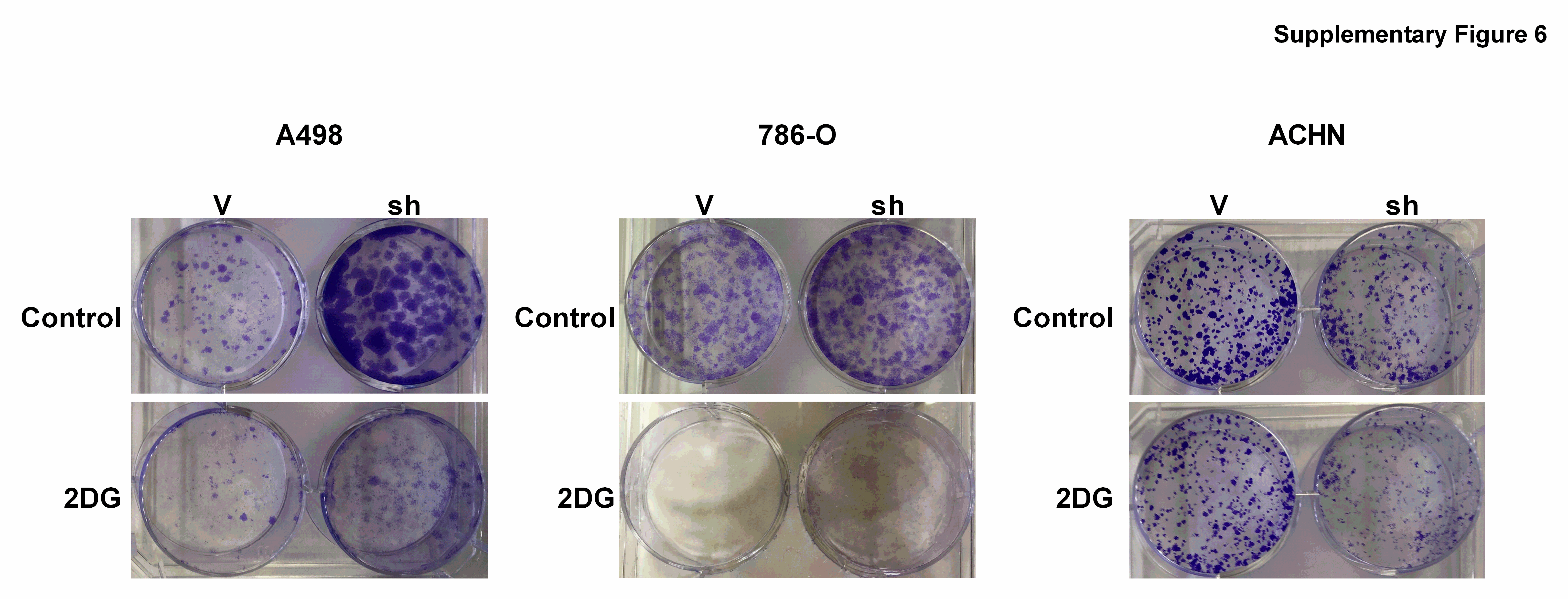


**Supplementary Figure 6. Clonal growth of control and MYBBP1A downregulated cells treated with 2DG (1mM).** The images are representative of 3 different experiments.


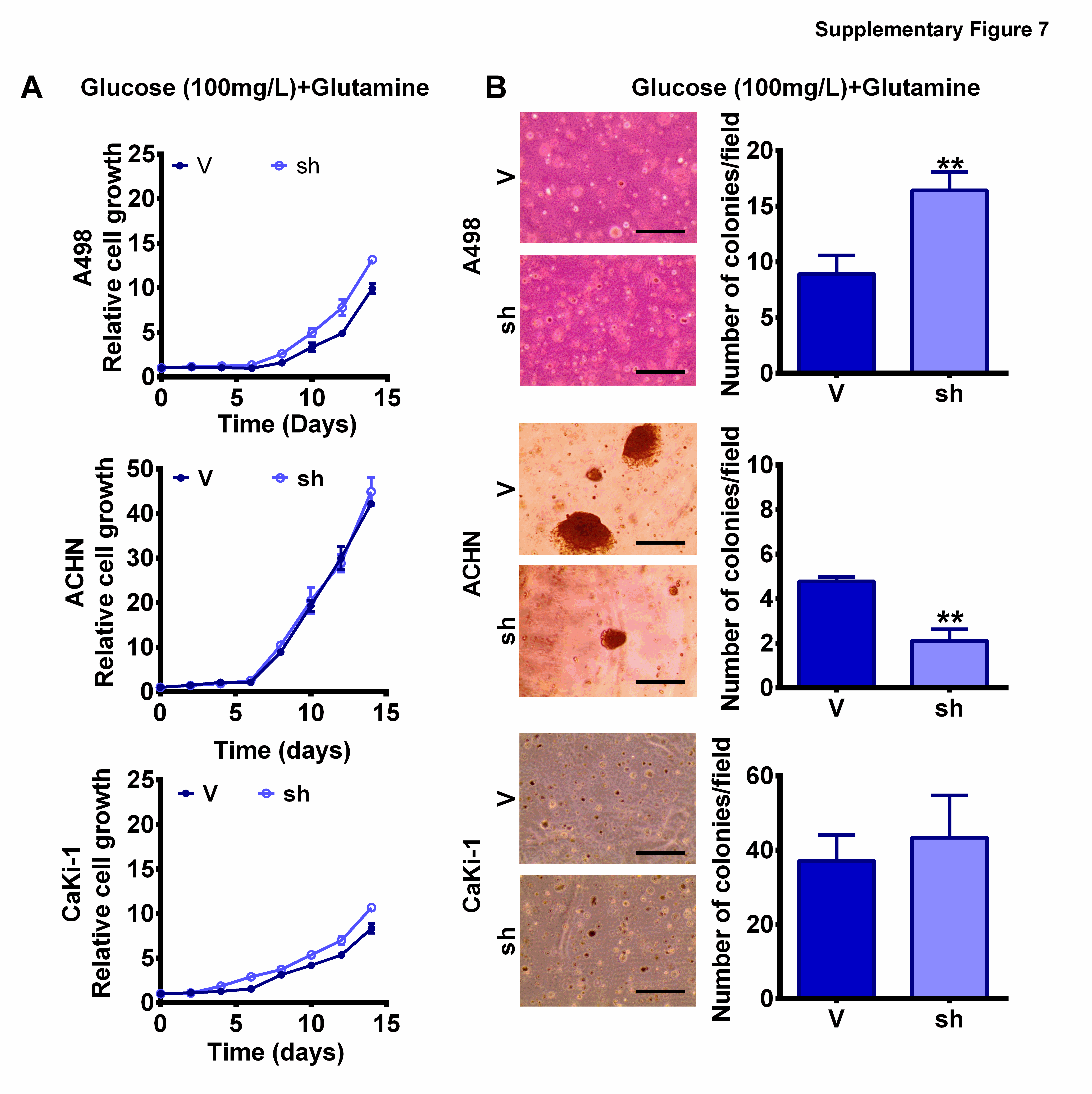


**Supplementary Figure 7. MYBBP1A reduction increases tumorigenic properties in c-MYB^+^ and pVHL^-^ cell lines under low glucose concentrations.** (A) Growth curves of A498, ACHN and CaKi-1 cells expressing the scramble vector (V) and cells with reduced levels of MYBBP1A (sh) in low glucose (100 mg/L) media. Representative experiment of three independent experiments performed in triplicate. Data represented are the mean±SD from triplicate samples.*p<0.05;**p<0.01;***p<0.001. (B) A498, ACHN and CaKi-1 cells expressing the scramble vector (V) or *MYBBP1A* shRNA (sh) were seeded in soft agar with 100 mg/L glucose. After 3-6 weeks, colonies were counted. Graphs shown the mean ±SD of three independent experiments performed in triplicate. Scale bars: 1cm= 625µm.*p<0.05; **p<0.01.


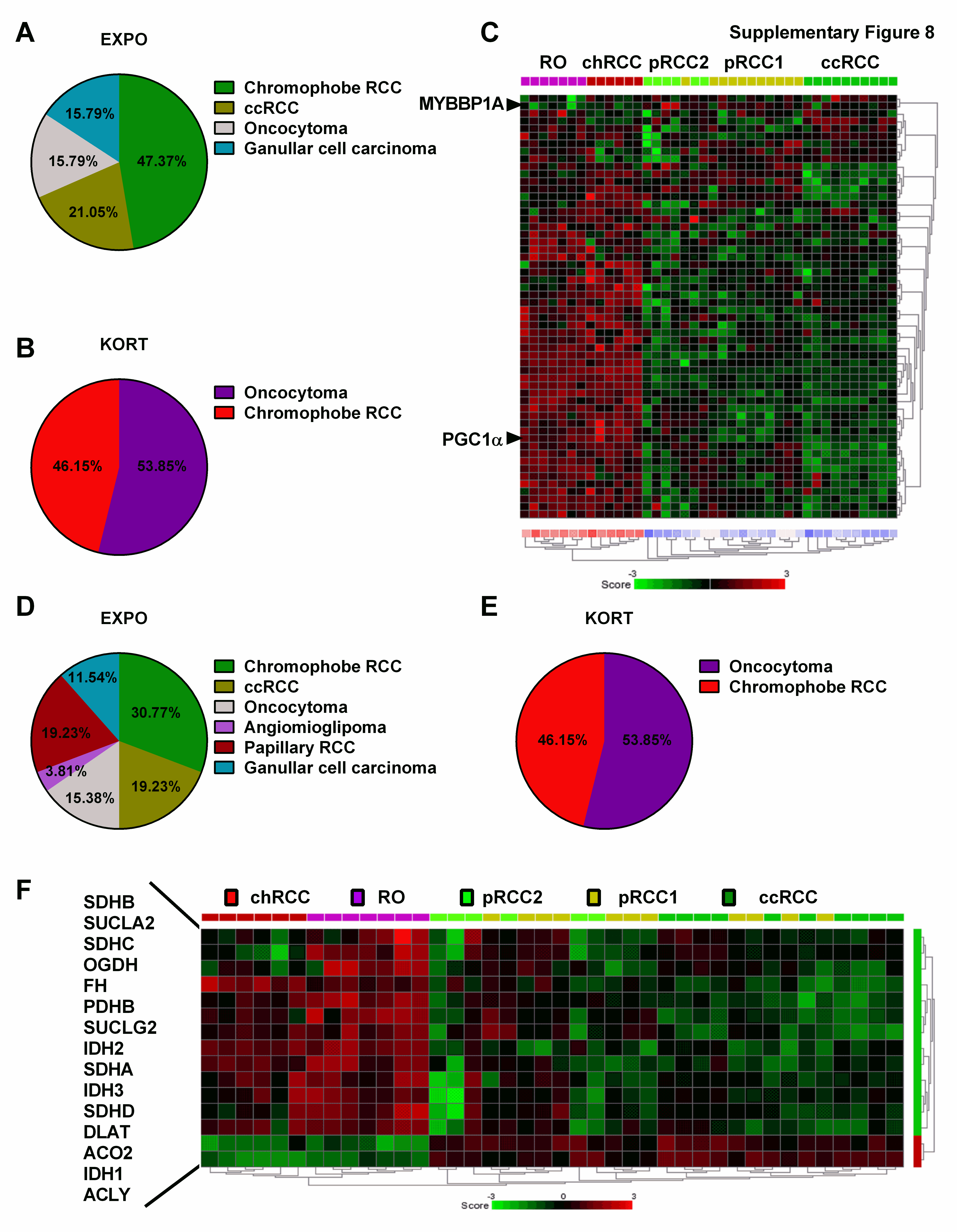


**Supplementary Figure 8. Analysis of *MYBBP1A* expression and its correlation with genes involved in metabolic pathways by different subtypes of RCCs.** (A) Subtypes of RCCs in the 8% of samples of EXPO database that have low *MYBBP1A* expression, high *PGC1α* expression and high expression of c-MYB target genes (frequency expressed in percentage). (B) Subtypes of RCCs in the 32% of samples of KORT database that have low *MYBBP1A* expression, high *PGC1α* expression and high expression of c-MYB target genes (frequency expressed in percentage). (C) Heat map of *MYBBP1A,* *PGC1α* and c-MYB target genes in the KORT database. Heat map was sorted by city-block distances. (D) Subtypes of RCCs in the 9% of samples of EXPO database that have low *MYBBP1A* expression and high expression of genes of the TCA cycle (frequency expressed in percentage). (E) Subtypes of RCCs in the 32% of samples of KORT database that have low *MYBBP1A* expression and high expression of genes of the TCA cycle (frequency expressed in percentage). (F) Heat map of genes of the TCA cycle that correlate positively (red bar) and negatively (green bar) with *MYBBP1A* in the KORT database. Heat map was sorted by Euclidean distance.

**Supplementary Table 1. Characteristics of cell lines.**

| **Cell line** | **Description** | **Mutation** | **c-MYB expression** |
| --- | --- | --- | --- |
| A498 | Human kidney carcinoma | VHL- | + |
| 786-0 | Human kidney carcinoma | VHL- , p53 | + |
| ACHN | Human kidney adenocarcinoma |  | - |
| CaKi-1 | Human kidney clear cell carcinoma |  | - |

VHL = Von Hippel Lindau

**Supplementary Table 2. Correlation between the expression of *MYBBP1A* and genes of the TCA cycle.**


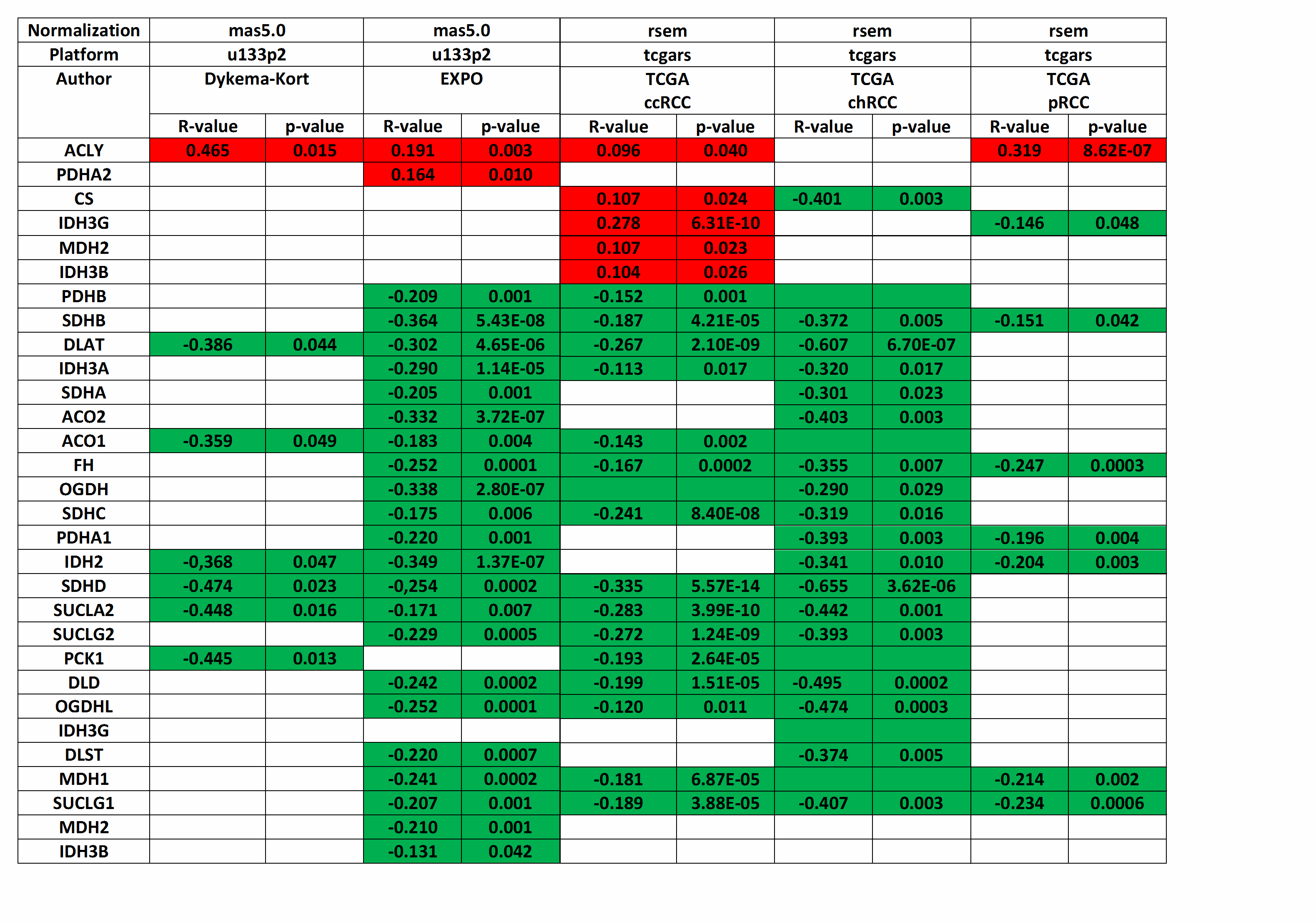

Supplement: Supplementary file 1 — Fig. S1 . Identification of an antisense fragment against MYBBP1A using a genetic loss‐of‐function screen in the absence of glucose. Fig. S2 . Reduction of MYBBP1A expression in renal, pancreas and liver tumors. Fig. S3 . Downregulation of MYBBP1A with a second shRNA. Fig. S4 . Expression of PGC1α in primary tumors and metastasis. Fig. S5 . Downregulation of MYBBP1A induces metabolic plasticity in 786‐O cell line with a second shRNA. Fig. S6 . Clonal growth of control and MYBBP1A downregulated cells treated with 2DG (1mM). Fig. S7 . MYBBP1A reduction increases tumorigenic properties in c‐MYB+ and pVHL‐ cell lines under low glucose concentrations. Fig. S8 . Analysis of MYBBP1A expression and its correlation with genes involved in metabolic pathways by different subtypes of RCCs. Table S1 . Characteristics of cell lines. Table S2 . Correlation between the expression of MYBBP1A and genes of the TCA cycle. [file MOL2-13-1519-s001.docx]
